# ATF4 promotes glutaminolysis and glycolysis in colorectal cancer by transcriptionally inducing SLC1A5: ATF4-dependent SLC1A5-mediated glycolysis

**DOI:** 10.3724/abbs.2024226

**Published:** 2024-12-17

**Authors:** Zengli Zhou, Shufang Ye, Jingyu Chen, Fei Dai, Luyi Chen, Ran Ye, Jianmei Zhang, Gefei Chen, Yanjiao Wang, Yangyang Liu

**Affiliations:** 1 Department of Gastroenterology Wenzhou Medical University Lishui Hospital Lishui People’s Hospital First Affiliated Hospital of Lishui University Lishui 323000 China; 2 Department of Gastroenterology the Second Affiliated Hospital of Zhejiang University School of Medicine Hangzhou 310009 China; 3 Department of General Practice Sir Run Run Shaw Hospital Zhejiang University School of Medicine Hangzhou 310020 China

**Keywords:** ATF4, SLC1A5, colorectal cancer, glycolysis

## Abstract

Glutaminolysis and glycolysis promote the malignant progression of colorectal cancer. The role of activating transcription factor 4 (ATF4) in solute carrier family 1 member 5 (SLC1A5)-mediated glutaminolysis and glycolysis remains to be elucidated. SLC1A5 and ATF4 expression levels are detected in colorectal cancer tissues. *ATF4* is knocked down or overexpressed to assess its role in cell viability, migration and invasion. *SLC1A5* is knocked down to evaluate its role in cell viability, migration, invasion, and metastasis and the metabolism of glutamine and glucose. The regulatory effect of the transcription factor ATF4 on SLC1A5 transcription and expression is determined using a luciferase reporter assay and chromatin immunoprecipitation (ChIP) techniques. Upregulated ATF4 and SLC1A5 expressions are observed in tumor tissue, which is positively correlated with the tumor, node, and metastasis (TNM) stages. *ATF4-*overexpressing SW480 cells show the increased cell viability, migration and invasion. Conversely, *ATF4* knockdown decreases the viability, migration and invasion of HCT-116 cells. *SLC1A5* knockdown inhibits viability, migration, invasion, and metastasis and the metabolism of glutamine and glucose in HT-29 cells, as well as the expressions of two key glycolytic enzymes, hexokinase 2 (HK2) and pyruvate kinase M2 (PKM2). The luciferase activity of the *SLC1A5* promoter is increased by *ATF4* overexpression. *SLC1A5* promoter enrichment is increased by anti-ATF4 antibody immunoprecipitation in *ATF4*-overexpressing colorectal cells, indicating that ATF4 targets SLC1A5 to promote glutamine and glucose metabolism in these cells. In summary, the ATF4/SLC1A5 axis plays a significant role in the progression of colorectal cancer by regulating glutamine metabolism and glycolysis.

## Introduction

Colorectal cancer is one of the most common digestive cancers in the human cancer spectrum; it has an annual incidence of 1.8 million cases and the second-highest mortality rate worldwide [ 1, 2]. Owing to the low rate of early diagnosis and the lack of effective therapeutic targets, the 5-year survival rate of affected patients is very low [ 3, 4]. Colorectal cancer is highly anabolic with increased glutaminolysis and glycolysis, which can induce a hypercatabolic state in patients. Mechanistically, both glutamine metabolism and glycolysis (the Warburg effect) play pivotal roles in promoting malignant proliferation and metastasis [5]. During treatment, cancer cells can exploit metabolic plasticity to resist metabolic and energetic blockade. Therefore, the inhibition of both glutaminolysis and glycolysis has become an emerging and promising drug discovery approach to combat cancer [6].

Notably, glutamine metabolism can interact with glycolysis to provide a carbon source for the glycolytic tricarboxylic acid cycle [ 7– 9]. In terms of mechanism, the intracellular signaling pathways of glycolysis and glutaminolysis are altered by oncogene activation and tumor suppressor gene inactivation [10]. The conversion of glutamine to glutamate is catalyzed by the mitochondrial glutaminase [11]. However, the molecular mechanisms by which glycolysis and glutaminolysis interact are not well defined.

Activating transcription factor 4 (ATF4), a basic leucine zipper transcription factor, is ubiquitously expressed throughout the body. Moreover, the ATF4 protein is induced in response to amino acid limitation or hypoxia to control life-death decisions. Once induced, ATF4 can directly control the transcription of several adaptive genes (metabolic or redox balance enzymes) and proapoptotic genes. On the other hand, ATF4 indirectly modulates autophagy and protein synthesis to influence survival [ 12 , 13]. Therefore, identifying the mechanisms that modulate the effects of ATF4 on cellular metabolism may reveal new targets for the treatment of colorectal cancer.

Solute carrier family 1 member 5 (SLC1A5), also known as ASCT2, is a transporter required for high-affinity uptake of glutamine by rapidly proliferating tumor cells. Glutamine uptake and subsequent glutaminolysis are vital for the activation of the mechanistic target of the rapamycin complex 1 (mTORC1) nutrient-sensing pathway, which can regulate cancer cell growth [14]. In colorectal cancer cells, SLC1A5 expression is upregulated to promote tumor cell proliferation, and the upregulation of SLC1A5 is significantly correlated with vascular invasion, depth of invasion, and distant metastasis [15]. Notably, ATF4 is considered a possible regulator of SLC1A5, and ATF4‐controlled survival mechanisms confer synthetic vulnerability to targeting glutaminolysis and glycolysis [16]. However, it is currently unclear whether ATF4 regulates the progression of colorectal cancer through SLC1A5 via glutaminolysis and glycolysis.

This study aimed to explore the functions of ATF4 and SLC1A5 in glutamine uptake and glycolysis, as well as their effects on the malignant migration and invasion of colorectal cancer cells. Elucidating the mechanism of colorectal cancer progression at the interaction level between glutamine metabolism and glycolysis will likely provide a potential target for colorectal cancer therapy.

## Materials and Methods

### Gene expression profiling interactive analysis (GEPIA) and tissue microarray analysis

The RNA sequencing data of patients with colon adenocarcinoma (COAD) were acquired from the GEPIA database, and the relative expression levels of *ATF4* and *SLC1A5* were screened, with *P* < 0.01 as the threshold for significance.

A human colorectal cancer tissue microarray (Shanghai Outdo Biotech, Shanghai, China) was used to detect the protein expressions of ATF4 and SLC1A5 using immunohistochemistry. The tissue samples were immunoreacted with primary antibodies against ATF4 (11815; Cell Signaling Technology, Danvers, USA) and SLC1A5 (8057S; Cell Signaling Technology), followed by incubation with horseradish peroxidase-labelled secondary antibodies (D-3004; Long Island Biotech, Shanghai, China). Immunoreactivity was assessed via the histochemical score (H score), which was calculated on the basis of the percentage of positively stained cells, by two independent investigators. Using the median H score as the cut-off point, the patients were classified into a high-expression (T-high) group or a low-expression (T-low) group.

### Cell culture

Human colorectal cancer cell lines (HCT-116, SW480, HT-29, SW620, and LOVO) and the normal human colonic epithelial cell line FHC were purchased from the Cell Bank of the of Biochemistry and Cell Biology, CAS (Shanghai, China) and were maintained in RPMI-1640 (Invitrogen, Carlsbad, USA) supplemented with 10% fetal bovine serum (Invitrogen) and 5000 U/mL penicillin/streptomycin (Invitrogen). The culture conditions were 5% CO _2_ and 37°C.

### Cell transfection

To overexpress *ATF4*, the *ATF4* gene sequence was cloned and inserted into the overexpression vector pcDNA3.1(+), referred to as oeATF4, by Generay Technologies (Shanghai, China), with the empty vector used as the control. To knock down *SLC1A5* or *ATF4*, SLC1A5-shRNA (shSLC1A5-1, 5′-GCTTCTTCAACTCCTTCAA-3′, shSLC1A5-2, 5′-CTGAGTTGATACAAGTGAA-3′, and shSLC1A5-3, 5′-GAAGGAATCAGTCATGTAA-3′) or ATF4-shRNA (ATF4-shRNA-1, 5′-CCGGGCCTAGGTCTCTTAGATGATTCTCGAGAATCATCTAAGAGACCTAGCTTTTT-3′) was constructed in pLKO.1 shRNA lentivirus by OBiO Technology Company (Shanghai, China). A lentiviral vector constructed with nonspecific shRNA was used as a negative control (shNC: 5′-TTCTCCGAACGTGTCACGT-3′). The recombinant lentiviral vectors were used to transduce 293T cells seeded in 96-well plates (3 × 10 ^3^ cells/well) with Lipofectamine 2000 (Invitrogen). The transfection reagents were removed after 6 h. Forty-eight hours post transduction, the recombinant lentivirus was collected and used to infect the colorectal cancer cell lines.

### Cell cycle and apoptosis analysis

A Cycle Test Plus DNA Reagent kit (BD Biosciences, Oxford, UK) was used to assess the cell cycle distribution. After incubation with propidium iodide (PI; 5 μL) for 10 min, flow cytometric analysis was performed on a FACStrak flow cytometer (Becton Dickinson, Heidelberg, Germany) with Modfit LTTM 2.0 software (Verity Software House, Cambridge, USA) to reveal the cell cycle distribution. An Annexin-V FITC Apoptotic Detection Kit I (BD Biosciences) was used to detect apoptosis according to the manufacturer’s instructions.

### Cell viability

An aliquot of cell counting kit-8 (CCK-8) solution (10 μL) was added to the supernatant of cultured cells, and the cells were incubated for 1 h. The cell viability was determined by the absorbance value at 450 nm using a SpectraMax M5 microplate reader (Molecular Devices, Sunnyvale, USA).

### Wound healing assay

Transfected colorectal cancer cells (5 × 10 ^5^ cells/well) were plated into a 6-well plate and cultured overnight until they reached approximately 100% confluence. After cell starvation for 6 h, the monolayer was scratched with a sterile 200-μL pipette tip to create an artificial homogenous wound. The wells of the plate were photographed at 0 h, 24 h, and 48 h, and the images were used to measure the wound healing capacity.

### Transwell assay

Transfected colorectal cancer cells (9 × 10 ^4^ cells) suspended in serum-free RPMI-1640 medium were placed in the upper chamber of a Matrigel-coated 24-well insert (BD Biosciences). Complete medium was added to the lower chamber. After cell culture for 24 h, the insert was removed, and the cells were fixed with methanol for 30 min. The cells that had invaded to the lower chamber were stained with 0.1 mg/mL crystal violet and photographed. The number of invasive cells was counted under a Nikon Eclipse Ti2-E microscope (Tokyo, Japan).

### Glutamine and glucose uptake assay

A glutamine assay kit (ab197011; Abcam, Cambridge, UK) was used to assess glutamine uptake following the manufacturer’s instructions. The 2-NBDG Glucose Uptake Assay Kit (K682-50; BioVision, San Francisco, USA) and flow cytometry, using the protocols recommended by the manufacturer, were used to measure glucose uptake.

### Measurement of ATP and lactate production

The cells (5 × 10 ^5^ cells/well) were seeded in a 6-well plate and subsequently treated with cell transfection reagents. Following a 48-h transfection period, the lactate release and ATP content in the cells were measured with an ATP assay kit and a lactic acid assay kit (Nanjing Jiancheng Bioengineering Institute, Nanjing, China), respectively, in accordance with the protocols recommended by the manufacturer.

### Quantitative real-time polymerase chain reaction (RT-qPCR)

Total RNA from colorectal cancer cells was extracted using Trizol reagent (Invitrogen). Following quantification by the ultraviolet spectrophotometric method, a total of 1 μg of RNA was reverse transcribed via the PrimeScript RT Reagent Kit (TaKaRa, Dalian, China). The cDNA products were amplified using Power SYBR Green PCR Master Mix (Thermo Fisher Scientific, Waltham, USA). The primers used were as follows: *SLC1A5*, 5′-ATCCATGGGCTCCTGGTACT-3′ (F), 5′-CACGCACTTCATCATCAGCG-3′ (R); *ATF4*, 5′-TACAACTGCCCTGTTCCC-3′ (F), 5′-GCTGAATGCCGTGAGAAG-3′ (R); and *β-actin*, 5′-AGGATTCCTATGTGGGCGAC-3′ (F), 5′-ATAGCACAGCCTGGATAGCAA-3′ (R). The expression of *β-actin* was used as the internal control.

### Western blot analysis

The harvested colorectal cancer cells were lysed on ice with lysis buffer (Roche, Basel, Switzerland) to isolate total protein. The quality of each protein sample was measured using the bicinchoninic acid method with a Protein Assay kit (Beyotime Biotechnology, Shanghai, China). The protein samples (25 μg) were separated by sodium dodecyl sulfate-polyacrylamide gel electrophoresis and then transferred to a PVDF membrane (Millipore, Billerica, USA). Nonspecific proteins in the membrane were blocked for 1 h using 5% nonfat milk. The target proteins in the membrane were probed by overnight incubation with primary antibodies against SLC1A5 (ab237704), hexokinase 2 (HK2) (ab209847), pyruvate kinase M2 (PKM2) (ab85555), ATF4 (ab184909), and β-actin (ab213262; all from Abcam). The following day, the membrane was incubated with a corresponding horseradish peroxidase-labelled secondary antibody (Beyotime Biotechnology; A0208 and A0216) at room temperature for 1 h. Visualization of the target proteins was achieved via an enhanced chemiluminescence detection system (Bio-Rad, Hercules, USA). β-Actin was served as a loading control.

### Dual-luciferase assay

ATF4-targeted *SLC1A5* promoter activity was analyzed by a dual-luciferase assay. In brief, the wild-type and mutant *SLC1A5* promoters were subcloned and inserted into the downstream region of the pGL3-Promoter firefly luciferase vector (Promega, Madison, USA). Colorectal cancer cells were seeded in 96-well plates and subsequently transfected with the *SLC1A5* promoter reporter and oeATF4 transfection using Lipofectamine 2000 (Invitrogen) at 37°C for 24 h. The luciferase activity of the SLC1A5 promoter reporter was measured with a Luciferase Assay System (Promega) following the protocols provided by the manufacturer.

### Chromatin immunoprecipitation (ChIP)

ATF4 binding to the SLC1A5 promoter was analyzed by a ChIP assay via a commercial assay kit (Millipore) according to the manufacturer’s instructions. In brief, colorectal cancer cells were co-transduced with oeATF4 or the control vector (empty vector) together with the wild-type or mutant *SLC1A5* promoter. After transfection for 24 h, the cells were fixed and sonicated. The lysates were then immunoprecipitated with an anti-ATF4 antibody (11815; Cell Signaling Technology). The *SLC1A5* promoter region enriched by ATF4 was detected by qPCR with the following primers: 5′-GGGATGTTACAACACCATG-3′ (F) and 5′-ACTACAGCCGCCAAAATA-3′ (R).

### Tumor xenograft

Colorectal cancer cells (5 × 10 ^6^ cells) expressing shSLC1A5 or shNC were injected into male nude mice (aged 4–5 weeks; *n* = 6 for each group; Shanghai Laboratory Animal Company, Shanghai, China) via the tail vein. For the *in vivo* metastasis assay, luciferase-labelled colorectal cancer cells (5 × 10 ^6^ cells) were injected into the tail vein of the mice to establish a lung metastasis model. For luciferase imaging, we injected d-luciferin-potassium salt into the abdominal cavity of the mice. Seven minutes after the injection, the mice were anaesthetized, and images were captured with an IVIS-100 system (Calliper Life Sciences, Hopkinton, USA). On day 21 after injection, the lung tissues were harvested from the animals to count metastatic nodules, and the tissue sections were stained with hematoxylin and eosin (H&E). All experiments involving animals were conducted in strict accordance with the protocol approved by the Ethics Committee of Wenzhou Medical University. The study was performed following the National Institutes of Health Guide for the Care and Use of Laboratory Animals.

### Hematoxylin and eosin (H&E)

All specimens were fixed in 4% paraformaldehyde solution for 24 h and embedded in paraffin and processed by standard histological processing techniques. Tissue sections (4-μm thick) were obtained from each sample. The hematoxylin and eosin results were observed under a light microscope at 40× magnification (BX51; Olympus, Tokyo, Japan) to observe the histology differences.

### Statistical analysis

GraphPad Prism version 8.4.2 software was used for data analysis. Data are presented as the mean ± standard deviation (SD). The Mann-Whitney U test was used to analyze the results. The overall survival rate of the patients was assessed using the Kaplan-Meier method and Cox proportional hazards regression model. Statistical significance between groups was determined using the log-rank test. All experiments were performed at least three times independently. A *P* value of less than 0.05 was considered to indicate a statistically significant difference.

## Results

### Upregulation of ATF4 and SLC1A5 is correlated with the metastasis of colorectal cancer

**Figure 1:**
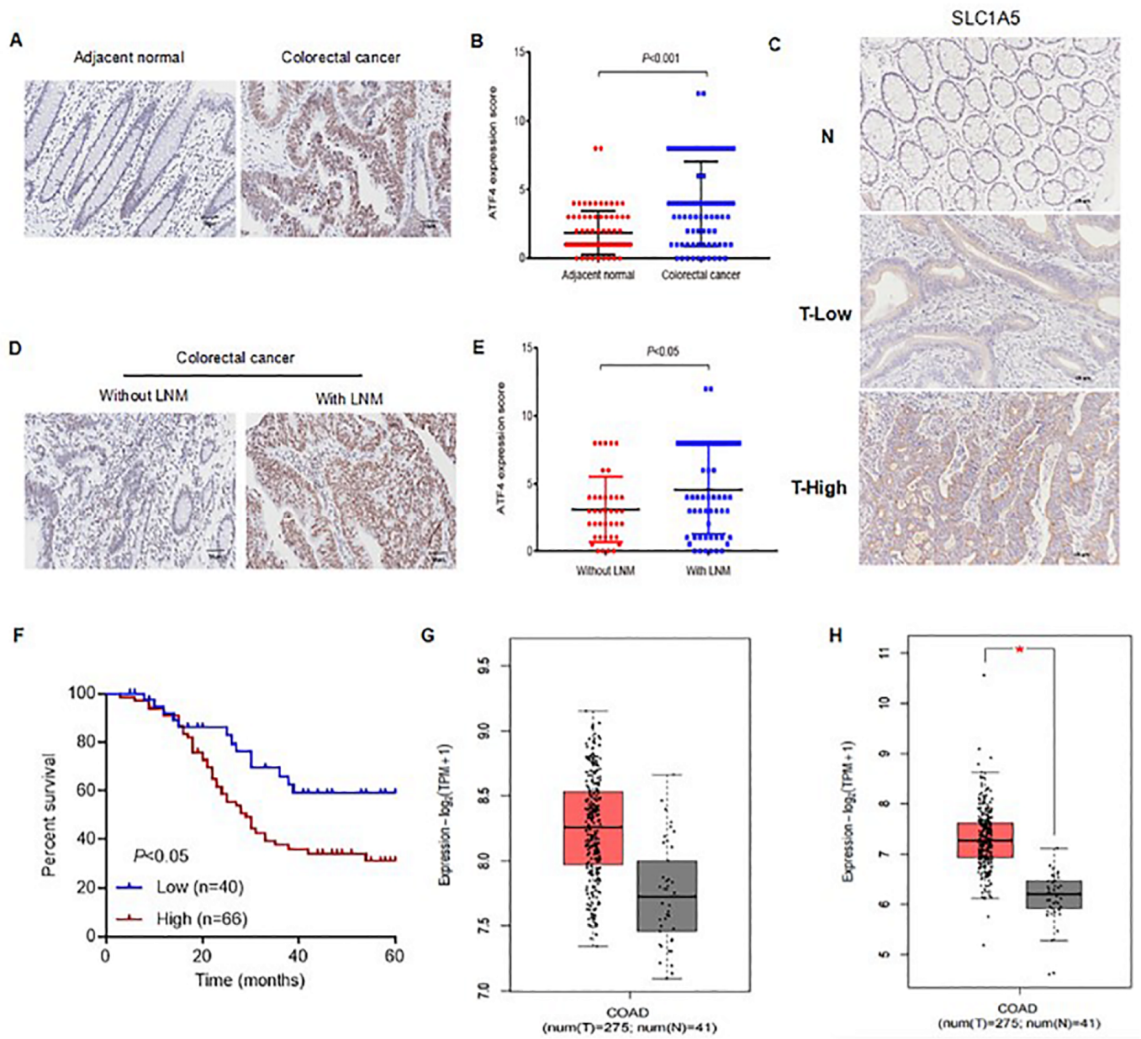
Figure 1 ATF4 and SLC1A5 expressions in colorectal cancer tissues and cell lines (A) ATF4 protein expression in colorectal cancer tissue was analyzed via immunohistochemistry in clinical colorectal cancer tissue microarrays. (B) The relative protein expression of ATF4 was quantitatively analyzed with ImageJ software. (C) SLC1A5 protein expression in colorectal cancer tissue was analyzed via immunohistochemistry in clinical colorectal cancer tissue microarrays. (D) Relative protein expression of ATF4 in clinical colorectal cancer tissues with or without lymph node metastasis (LNM). (E) The relative protein expression of ATF4 in colorectal cancer tissues with or without LNM was quantitatively analyzed with NIH ImageJ software. (F) Kaplan-Meier plot of the cumulative probability of remaining event free for patients with colorectal cancer. * P < 0.05. (G) ATF4 mRNA expression in colon adenocarcinoma (COAD) samples in the Gene Expression Profiling Interactive Analysis (GEPIA) database was determined. (H) SLC1A5 mRNA expression in COAD samples in the GEPIA database was determined.

Compared with those in paracancerous tissues (adjacent normal tissues), increased expression levels of ATF4 ( Figure 1A,B) and SLC1A5 ( Figure 1C) were detected in colorectal cancer tumor tissues. Higher ATF4 expression was detected in colorectal cancer patients with lymph node metastasis compared with those without lymph node metastasis ( Figure 1D,E). High ATF4 expression was correlated with tumor node metastasis (TNM) stage and lymph node metastasis ( Table 1), whereas high SLC1A5 expression was correlated with tumor size, TNM classification, and lymph node metastasis ( Table 2). Furthermore, the relative expression of SLC1A5 could be used to predict the overall survival of patients with colorectal cancer ( Figure 1F). As expected, the expression levels of *ATF4* ( Figure 1G) and *SLC1A5* ( Figure 1H) were higher in the COAD tissues than in the matched normal tissues, according to the GEPIA data.

**Table:** **Table 1** Correlations between ATF4 protein expression and clinicopathological parameters in patients with CRC

Clinicopathological parameter	Protein expression of ATF4		*P* value
High ( *n* = 56)	Low ( *n* = 50)	
Gender				0.193
Male	26	17		
Female	30	33	
Age (years)				0.134
< 60	21	26		
≥ 60	35	24	
Tumor size (cm)				0.000
≤ 4	13	33		
> 4	43	17	
TNM classification				0.000
I/II	12	37		
III/IV	44	13	
T Stage				0.020
T1	9	11		
T2	13	20	
T3	18	11	
T4	16	8	
Lymph node metastasis				0.000
Yes	36	15		
No	20	35	

Differences between groups were determined by the Chi-square test.

**Table:** **Table 2** Correlation between SLC1A5 protein expression and clinicopathological parameters in patients with CRC

Clinicopathological parameter	Protein expression of SLC1A5		*P* value
High ( *n* = 66)	Low ( *n* = 40)	
Gender				0.364
Male	29	14		
Female	37	26	
Age (years)				0.610
< 60	28	19		
≥ 60	38	21	
Tumor size (cm)				0.002
≤ 4	21	25		
> 4	45	15	
TNM classification				0.005
I/II	20	29		
III/IV	46	11	
T stage				0.045
T1	12	8		
T2	16	17	
T3	21	8	
T4	17	7	
Lymphnode metastasis				0.035
Yes	38	13		
No	28	27		

Differences between groups were determined by the Chi-square test.

### ATF4 promotes the viability, migration, and invasion of CRC cells

**Figure 2:**
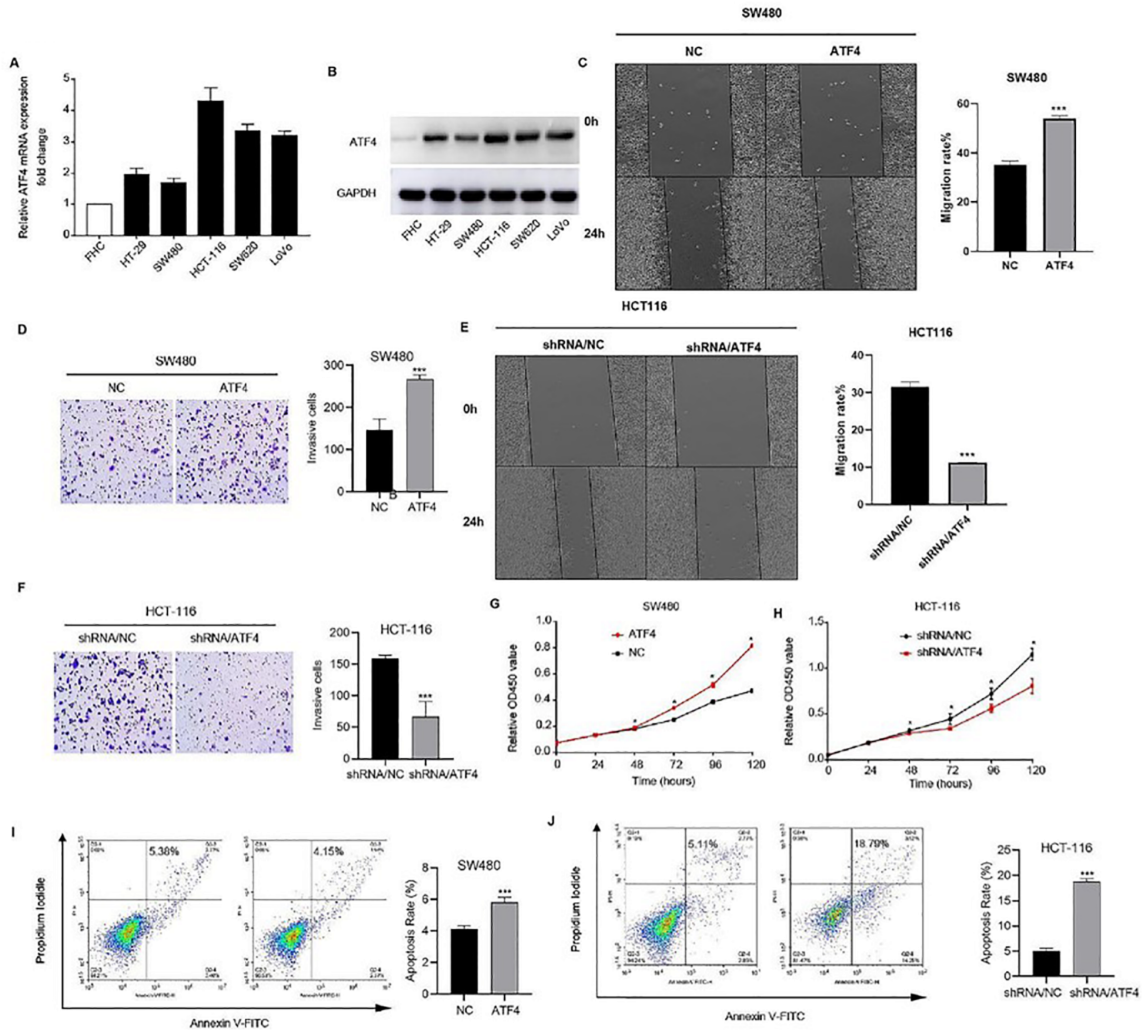
Figure 2 *ATF4* knockdown inhibits the viability, migration, invasion, and metastasis of colorectal cancer cells (A,B) The relative mRNA (A) and protein (B) expression levels of ATF4 were examined in colorectal cancer cell lines. (C,D) Wound healing assay (C) and transwell assay (D) of ATF4-transfected SW480 cells. (E,F) HCT-116 cells transfected with ATF4 shRNA were assayed via a wound healing assay (E) and a transwell assay (F). (G,H) The cell viability of ATF-overexpressing SW480 cells (G) and ATF-knockdown HCT-116 cells (H) was determined with a Cell Counting Kit-8 assay. (I,J) Apoptosis in ATF-overexpressing SW480 cells (I) and ATF-knockdown HCT-116 cells (J) was detected by Annexin-V-FITC/propidium iodide staining. *P < 0.05, *** P < 0.001.

The relative mRNA expression ( Figure 2A) and protein expression ( Figure 2B) of ATF4 were detected in various colorectal cancer cell lines. Notably, highly invasive colorectal cancer cell lines (SW620, HCT-116, and LoVo) presented relatively high expression of ATF4 compared with minimally invasive colorectal cancer cell lines (SW480 and HT-29). In addition, *ATF4*-overexpressing SW480 cells presented increased migration ( Figure 2C) and invasion ( Figure 2D), as assessed by wound healing and transwell assays, respectively. Furthermore, HCT-116 cells with *ATF4* knockdown exhibited decreased migration ( Figure 2E) and invasion ( Figure 2F).

Our results further revealed that *ATF4* overexpression significantly increased the proliferation ( Figure 2G) and survival ( Figure 2I) of SW480 cells, whereas ATF4 knockdown significantly decreased the proliferation ( Figure 2H) and survival ( Figure 2J) of HCT-116 cells. Cell cycle analysis revealed an increased percentage of S-phase cells among ATF4-overexpressing SW480 cells ( Supplementary Figure S1A) and a decreased percentage of S-phase cells among ATF4-inhibited HCT116 cells ( Supplementary Figure S1B). Collectively, these findings suggested that ATF4 affects colorectal cancer cell viability, migration, and invasion.

### SLC1A5 promotes the viability, migration, invasion, and metastasis of colorectal cancer

**Figure 3:**
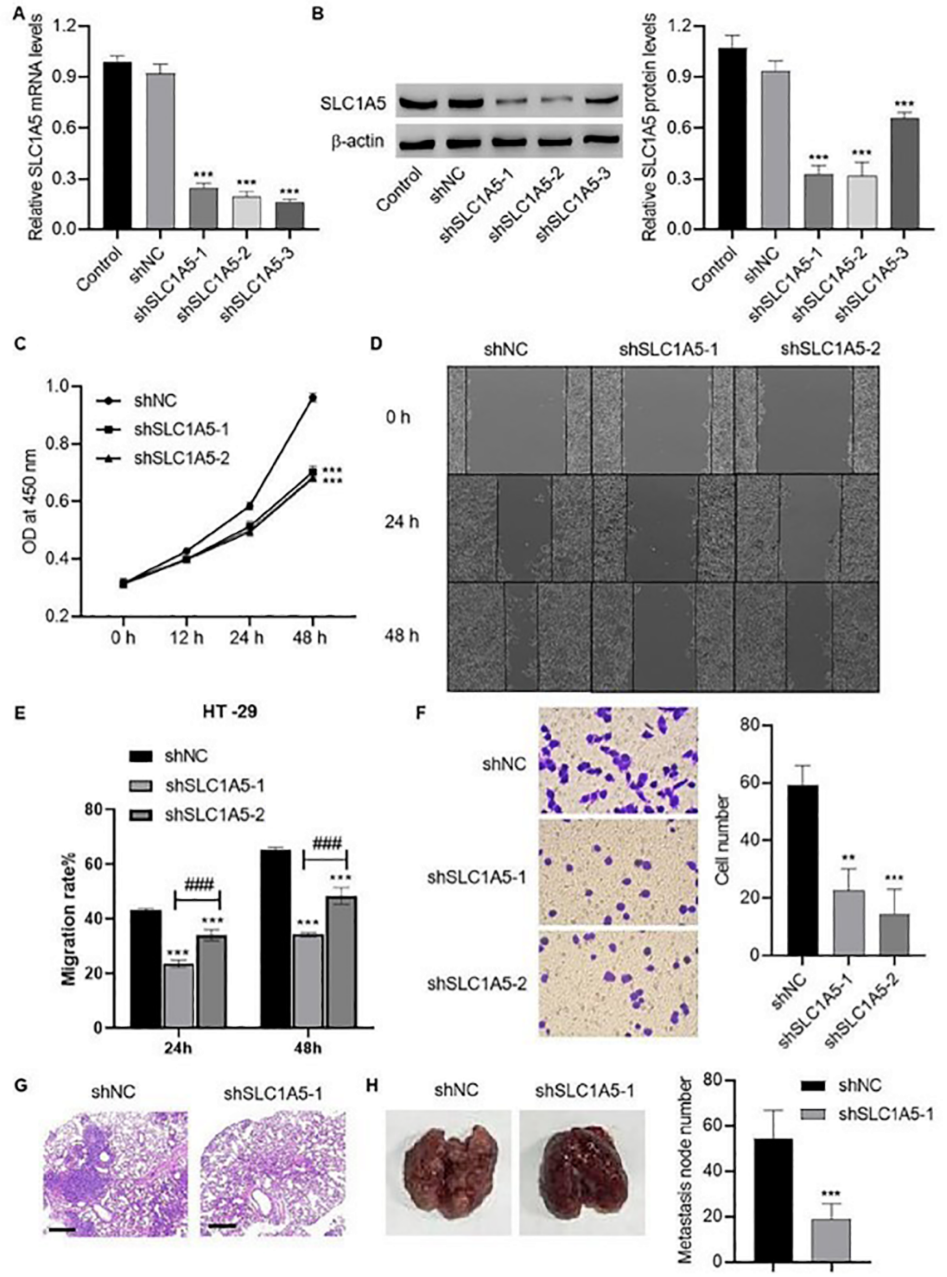
Figure 3 *SLC1A5* knockdown inhibits the viability, migration, invasion, and metastasis of colorectal cancer cells (A,B) SLC1A5 mRNA (A) and protein (B) expression was examined in HT-29 cells expressing SLC1A5 shRNA-1, -2, or -3 and compared with that in cells expressing nonspecific shRNA as the negative control (shNC). (C–F) Cell viability (C), wound healing (D,E), and invasion (F) of HT-29 cells expressing shSLC1A5-1, -2, or shNC. BALB/c nude mice were injected with HT-29 cells expressing shSLC1A5-1 or shNC. (G,H) Lung tissues were isolated for H&E staining (G) and metastatic node counting (H). Scale bar: 500 μm. **P < 0.01, ***P < 0.001 vs shNC; ###P < 0.001, shSLC1A5-1 vs shSLC1A5-2.

We knocked down *SLC1A5* in HT-29 cells through shRNA lentiviral vectors to investigate the effect of *SLC1A5* knockdown on the malignant biological characteristics of HT-29 cells. Compared with shNC, shSLC1A5-1, -2, and -3 effectively decreased *SLC1A5* mRNA levels ( Figure 3A). Accordingly, the SLC1A5 protein expression level was also reduced by shSLC1A5-1, -2, and -3 ( Figure 3B). shSLC1A5-1-knockdown cells and shSLC1A5-2-knockdown cells were selected for further analysis of cell characteristics. Cell viability was reduced following *SLC1A5-1* knockdown and *SLC1A5-2* knockdown ( Figure 3C). In addition, cell migration was suppressed in shSLC1A5-1-knockdown cells and shSLC1A5-2-knockdown cells after 48 h ( Figure 3D). The migration distance of these cells was measured ( Figure 3E), and cell invasion was inhibited by *SLC1A5-1* knockdown and *SLC1A5-2* knockdown ( Figure 3F). shSLC1A5-1-knockdown cells were subsequently injected into nude mice to assess tumor metastasis. H&E staining of the lungs revealed metastatic tumor nodes ( Figure 3G). A comparison of metastatic nodes in lung tissues from shNC-injected mice and shSLC1A5-1-injected mice was performed ( Figure 3H). Statistical analysis revealed that the number of metastatic nodes in shSLC1A5-1-injected mice was significantly lower than that in shNC-injected mice ( Figure 3H).

### ATF4 can transcriptionally promote the expression of SLC1A5

**Figure 4:**
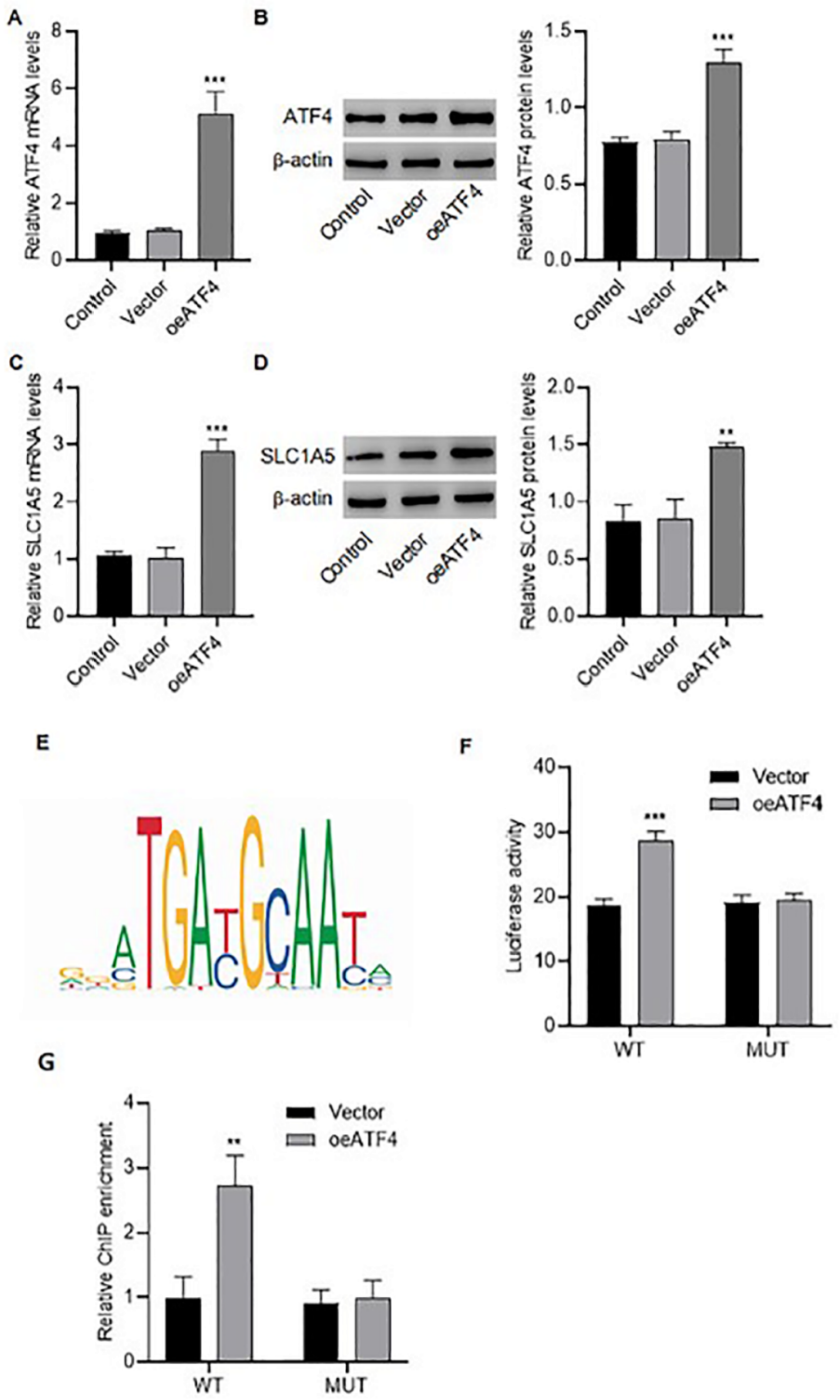
Figure 4 ATF4 promotes SLC1A5 expression (A–D) ATF4 (A,B) and SLC1A5 (C,D) expressions in SW480 cells transfected with an ATF expression vector (oeATF4) or a blank vector. (E) The binding sites of ATF4 and SLC1A5 were predicted by JASPAR. (F) Luciferase activity of the wild-type (WT) or mutant (MUT) SLC1A5 promoter in SW480 cells transfected with oeATF4 or the empty vector. (G) The enrichment of the SLC1A5 promoter in anti-ATF4 immunoprecipitates from cells transfected with the WT or MUT SLC1A5 promoter and the oeATF4 or blank vector was determined by qPCR. **P < 0.01, ***P < 0.001 vs vector.

**Figure 5:**
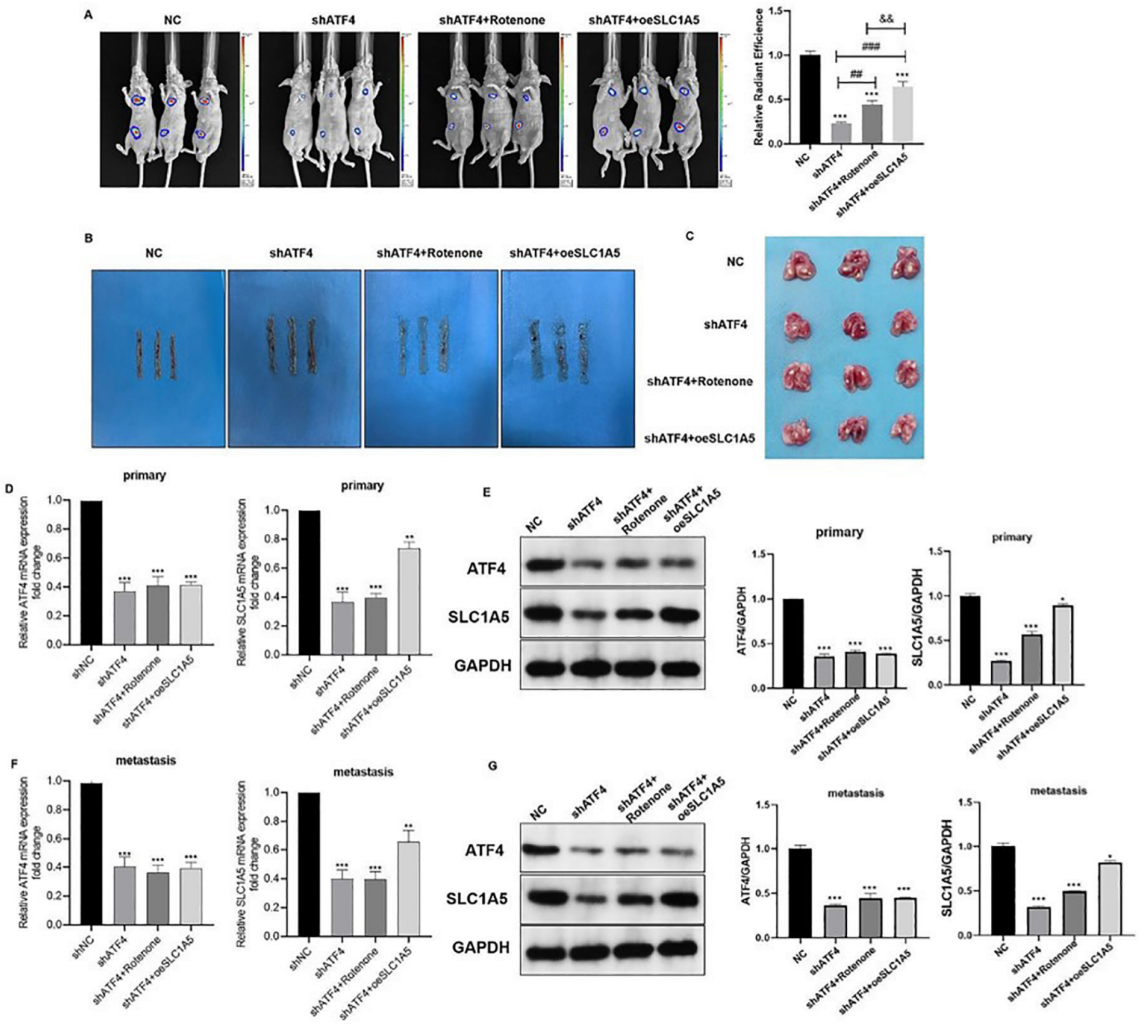
Figure 5 ATF4 promotes the growth and metastasis of colorectal cancer by targeting SLC1A5 *in vivo* (A) Representative images were taken on the 14th day (left panel), and quantification of metastatic cells in the mouse body was performed via bioluminescence analysis (right panel). (B,C) Mice were sacrificed, and colorectal tissue and lungs were removed from the mice. Images showing metastases in the lungs. (D,E) ATF4 and SLC1A5 expressions in primary lesions in colorectal tissue. (F,G) ATF4 and SLC1A5 expressions in lung metastases. *P < 0.05, **P < 0.01, ***P < 0.001 vs shNC; ##P < 0.01, ###P < 0.001 vs shATF4; &&&P < 0.001 vs shATF4 + Rotenone.

ATF4 was overexpressed in SW480 cells ( Figure 4A,B). In the *ATF4*-overexpressing cells, the mRNA and protein expression levels of SLC1A5 were increased ( Figure 4C,D). To investigate the effect of ATF4 on SLC1A5 expression, the binding sites of the ATF4 and SLC1A5 promoters were predicted by JASPAR ( Figure 4E) [17]. The *ATF4*-overexpression vector and luciferase SLC1A5 promoter reporter were cotransfected into SW480 cells. *ATF4* overexpression increased the luciferase activity of the *SLC1A5* promoter but had no effect on the activity of the mutant *SLC1A5* promoter ( Figure 4F). According to the ChIP assay results, the enrichment of SLC1A5 mRNA was increased in *ATF4*-overexpressing SW480 cells, as shown by immunoprecipitation with an anti-ATF4 antibody ( Figure 4G). This study’s findings from both the luciferase reporter assay and ChIP analysis demonstrated that ATF4 could transcriptionally regulate SLC1A5. We also developed *ATF4*-knockdown and *SLC1A5*-overexpressing CRC tumor xenografts to determine whether ATF4 regulates the growth and metastasis of colorectal cancer through the modulation of SLC1A5 expression ( Figure 5). Bioluminescence analysis showed a quantitative reduction in metastatic cells after *ATF4* knockdown, which was then rescued by overexpression of SLC1A5 ( Figure 5A). The metastatic tumor nodes of lungs were revealed ( Figure 5B,C). Then we detected the mRNA and protein levels of ATF4 and SLC1A5 and found that the expression of ATF4 and SLC1A5 in the shATF4 group were significantly reduced compared to the shNC group ( Figure 5D–G).

### SLC1A5 promotes glutamine and glucose metabolism in colorectal cancer

**Figure 6:**
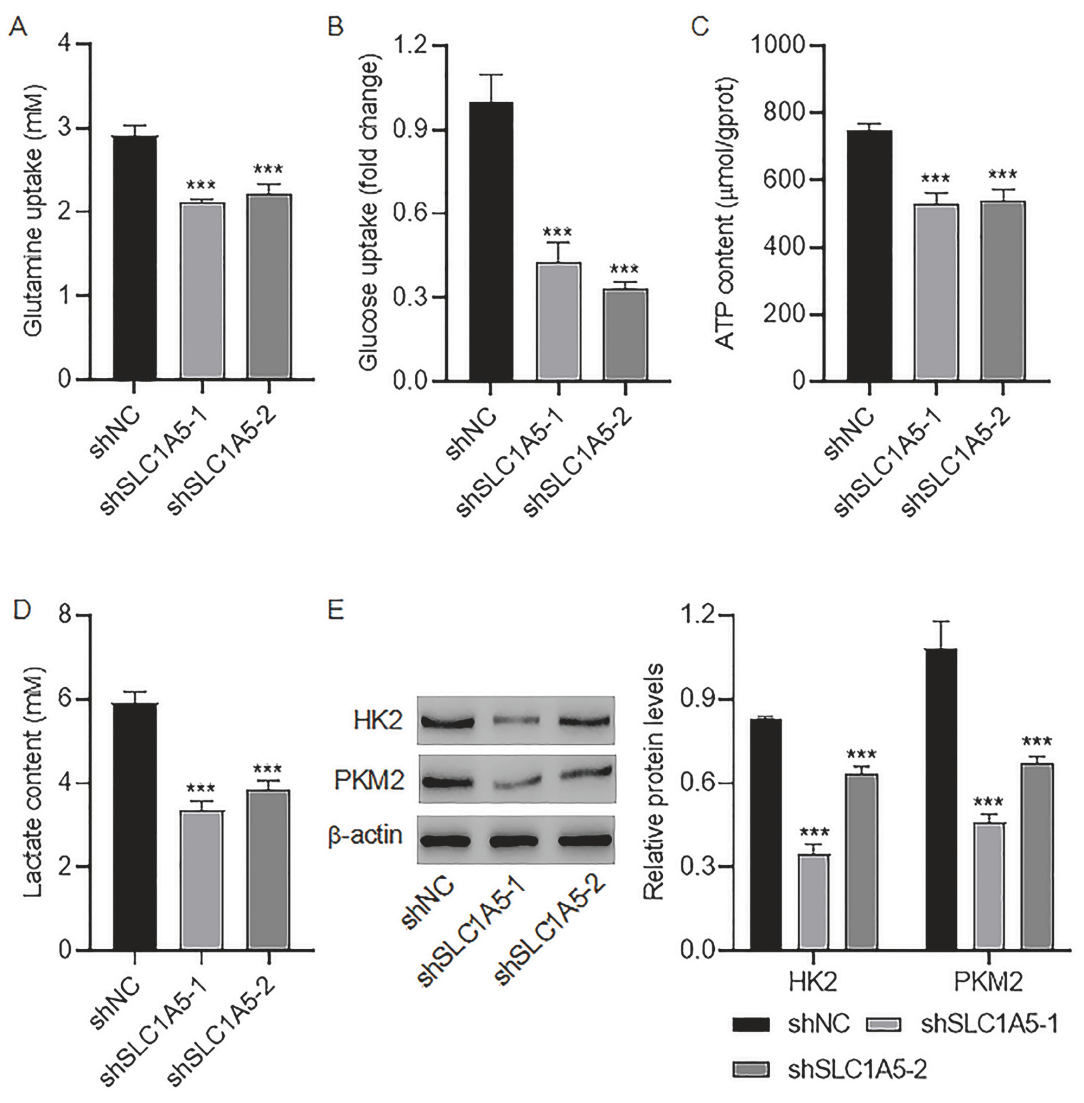
Figure 6 *SLC1A5* knockdown inhibits glutamine and glucose metabolism in colorectal cancer cells (A–E) Glutamine uptake (A), glucose uptake (B), ATP level (C), lactate content (D), and expression of HK2 and PKM2 (E) in colorectal cancer cells expressing shSLC1A5-1, -2, or shNC. ***P < 0.001 vs shNC.

To explore the potential mechanism by which *SLC1A5* knockdown exerts its antitumor effects on colorectal cancer, we examined glutamine and glucose metabolism in shSLC1A5-1-knockdown cells and shSLC1A5-2-knockdown cells. Figure 6A shows that glutamine uptake was reduced by shSLC1A5-1 and -2. Similarly, glucose uptake, ATP production, and lactate content were also suppressed by shSLC1A5-1 and -2 ( Figure 6B–D). The expression of two rate-limiting enzymes in the glycolytic pathway, PKM2 and HK2, was reduced by shSLC1A5-1 and -2 ( Figure 6E). These findings suggested that *SLC1A5* knockdown not only suppresses glutamine metabolism but also restricts aerobic glycolysis.

### ATF4 promotes glutamine and glucose metabolism in colorectal cancer cells by targeting SLC1A5

**Figure 7:**
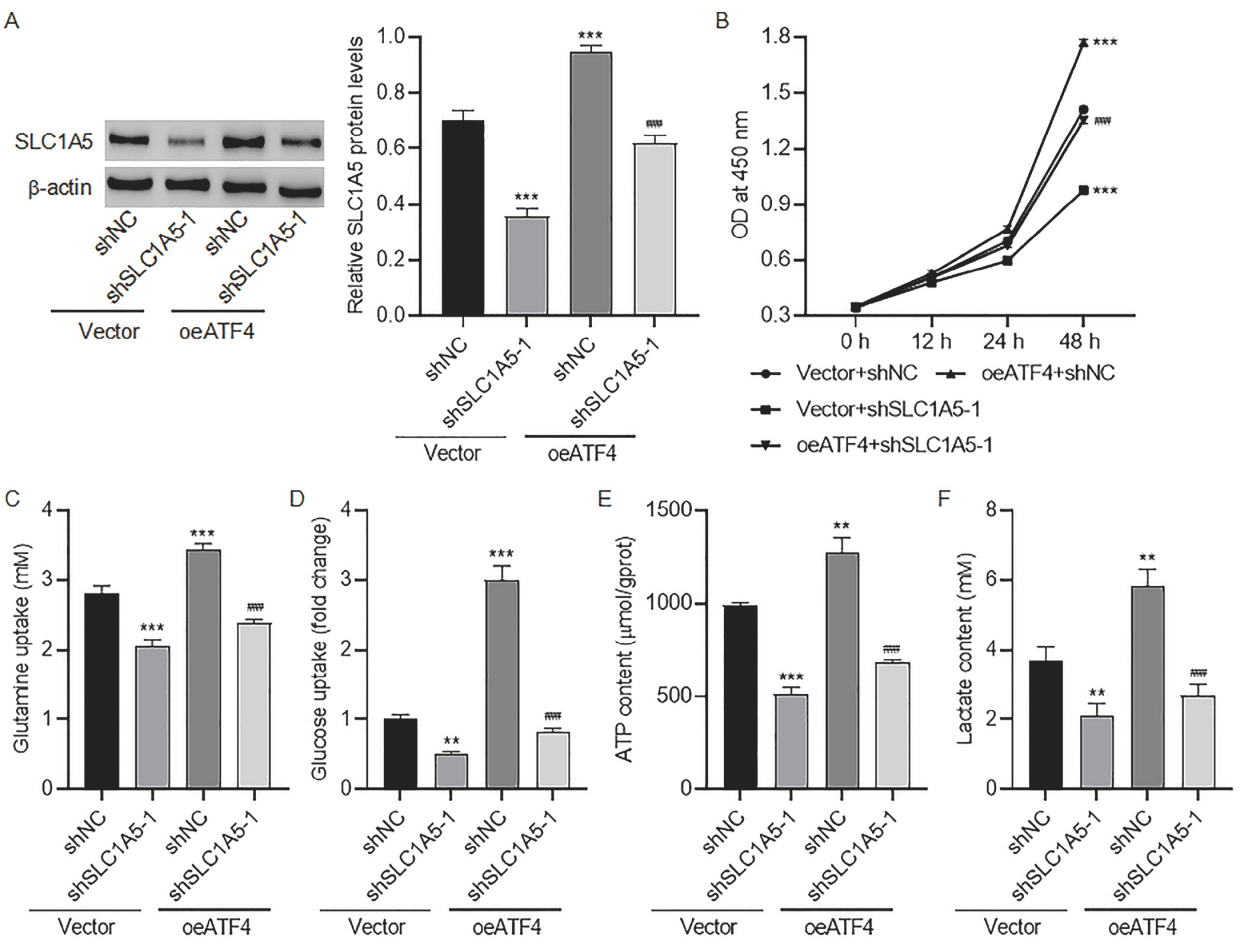
Figure 7 ATF4 promotes glutamine and glucose metabolism in colorectal cancer cells by targeting SLC1A5 (A–F) SLC1A5 expression (A), cell viability (B), glutamine uptake (C), glucose uptake (D), ATP (E), and lactate content (F) in colorectal cancer cells expressing shSLC1A5 and/or oeATF4. **P < 0.01, ***P < 0.001 vs vector + shNC. ###P < 0.001 vs oeATF4 + shNC.

**Figure 8:**
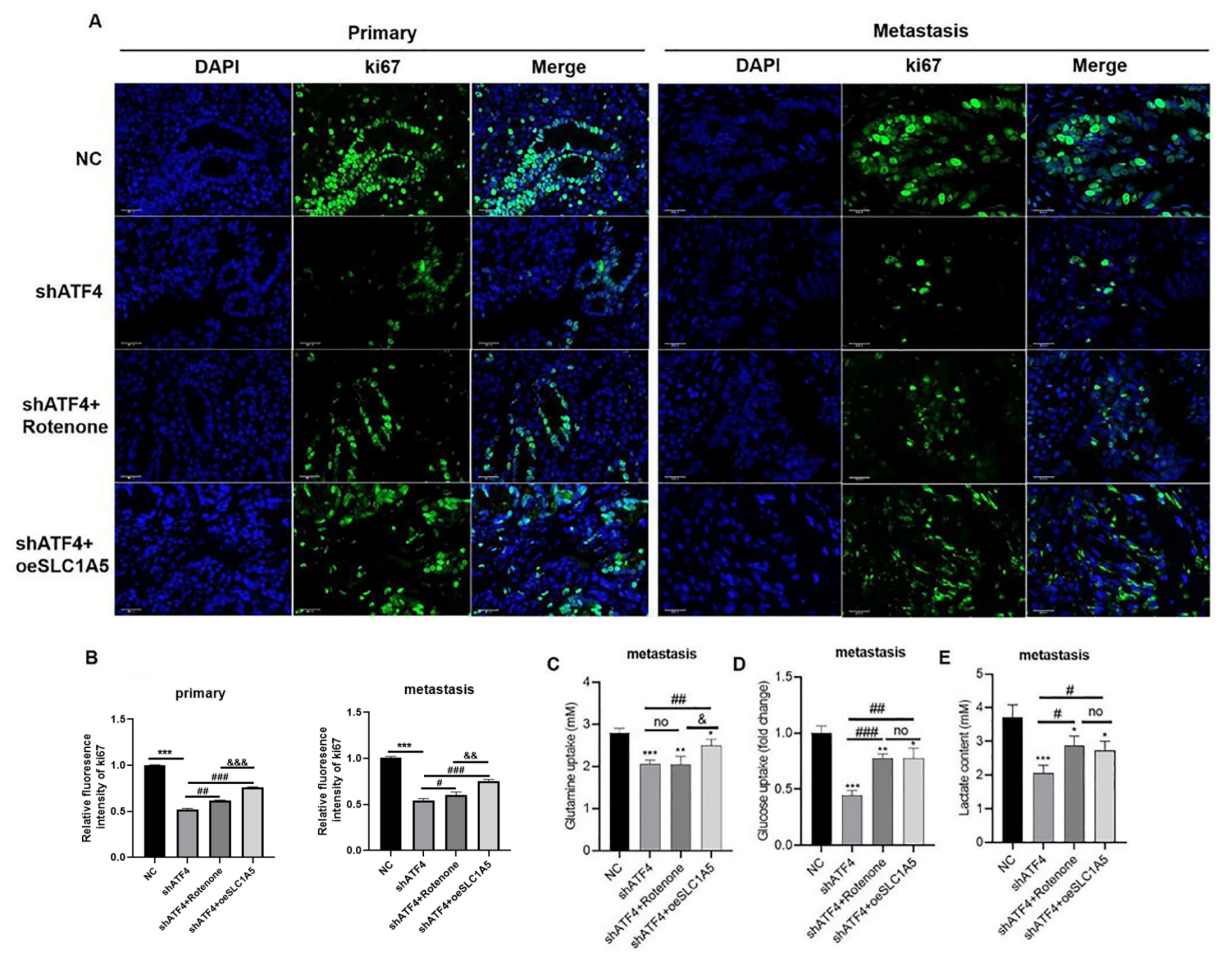
Figure 8 ATF4 promotes glutaminolysis and glycolysis in colorectal cancer by targeting SLC1A5 *in vivo* (A,B) Immunofluorescence of Ki67 in primary and metastatic lesions. (C) Glutamine uptake. (D) Glucose uptake. (E) Lactate content of metastases in the lungs. *P < 0.05, **P < 0.01, ***P < 0.001 vs shNC; #P < 0.05, ##P < 0.01, ###P < 0.001 vs shATF4; &P < 0.05, &&P < 0.01, &&&P < 0.001 vs shATF4 + Rotenone.

Given that ATF4 has regulatory effects on SLC1A5 expression, we investigated the impact of their regulatory interactions on glutamine and glucose metabolism. SLC1A5 expression was decreased by shSLC1A5 transfection, but this reduction was partially reversed by ATF4 overexpression ( Figure 7A). In addition, compared with shSLC1A5-knockdown cells, colorectal cancer cells cotransfected with oeATF4 and shSLC1A5 presented increased cell viability ( Figure 7B). Similarly, glutamine uptake ( Figure 7C), glucose uptake ( Figure 7D), ATP production ( Figure 7E), and lactate content ( Figure 7F) were significantly greater in colorectal cancer cells co-transfected with oeATF4 and shSLC1A5 than in SLC1A5-knockdown cells. Collectively, these findings indicated that ATF4 promotes glutamine and glucose metabolism in colorectal cancer cells by targeting SLC1A5. We also developed *ATF4*-knockdown and *SLC1A5*-overexpressing CRC tumor xenografts to determine whether ATF4 promotes glutamine and glucose metabolism in colorectal cancer cells by targeting SLC1A5 ( Figure 8). The results showed that the levels of glutamine, glucose, and lactate in the metastatic lesion were negatively correlated with *ATF4* knockdown, and were subsequently rescued with *SLC1A5* overexpression or Rotenone treatment ( Figure 8).

## Discussion

Glutamine metabolism and aerobic glycolysis are correlated with the aggressive behavior of tumors, as well as with resistance to radiotherapy and chemotherapy. Therefore, targeting tumor metabolism may represent a promising direction for the development of therapeutic strategies [18]. Our current study confirmed the critical role of the ATF4/SLC1A5 axis in regulating glutamine uptake and glycolysis as well as in tumor cell proliferation and metastasis. Consequently, the ATF4/SLC1A5 axis should be considered a potential therapeutic target for the treatment of colorectal cancer.

Colorectal cancer cells are metabolically reprogrammed to support high rates of proliferation, growth, survival, invasion, metastasis, and resistance to cancer treatments. As a stress-induced transcription factor, ATF4 is part of the amino acid response pathway GCN2/eIF2α/ATF4 and plays a crucial role in promoting the proliferation, metastasis, and chemoresistance of colorectal cancer cells [ 19– 21]. In non-small cell lung cancer, activated ATF4 is responsible for the activation of SLC1A5, thereby promoting glutamine consumption and metabolism [22]. In proliferating Drosophila cells, ATF4 is believed to regulate the expression of multiple enzymes involved in the glycolytic pathway [ 23, 24]. Other studies have shown that ATF4 plays an important role in regulating glucose and lipid metabolic homeostasis in hepatocytes and adipocytes [25]. ATF4 also promotes catabolic glycolysis and glutaminolysis, as well as oxidative phosphorylation, and thereby provides precursors and energy for anabolic pathways in CD4 ^+^ T cells [12]. Our data suggest that the ATF4/SLC1A5 signaling axis contributes to glutamine metabolism and glycolysis, which are necessary for the proliferation and metastasis of colorectal cancer cells.

Previous research has demonstrated that SLC1A5 can promote the survival, growth, and migration of colorectal cancer cells [ 15, 26, 27]. ATF4 binds to the ATF binding site located between -700 and -447 in the human SLC1A5 promoter [28]. The downregulation of ATF4 by ATF4 RNA interference blocked the upregulation of the SLC1A5 promoter [29]. Our current study also revealed that two glycolytic pathway-related rate-limiting enzymes, HK2 and PKM2, are regulated by SLC1A5, which further supports the potential role of ATF4/SLC1A5-mediated glutamine metabolism and glycolysis in colorectal cancer. Mutations in KRAS codon 12 are frequently observed (nearly 50%) in metastatic colorectal cancer patients with a poor prognosis, and abnormal RAS proteins have been considered undruggable until recently [30]. Moreover, a novel monoclonal antibody targeting ASCT2 has been demonstrated to reduce KRAS-mutated human colorectal cancer cell growth in a human xenograft model [31]. These findings suggest the potential of targeting the ATF4/SLC1A5 axis in the treatment of KRAS-mutated human colorectal cancer patients.

Interestingly, although SLC1A5 functions as a glutamine transporter, SLC1A5 knockdown has a greater inhibitory effect on colorectal cancer cell growth than does glutamine depletion [15], suggesting that the ATF4/SLC1A5 axis may modulate the malignant phenotype of tumor cells, independent of glutamine metabolism. However, the potential roles of the ATF4/SLC1A5 axis in properties other than glutamine metabolism and aerobic glycolysis in tumor cells require further investigation.

Notably, during periods of stress, a wide range of adaptive genes can be regulated by ATF4. Under persistent stress, ATF4 promotes the induction of apoptosis [32]. Research has indicated that posttranslational modifications and other epigenetic mechanisms can modulate the balance between the anti- and prosurvival effects of ATF4 [ 16, 33]. Taken together, these findings suggest that strategies targeting the specific increase in ATF4 degradation or the inhibition of its transcriptional activity need to be carefully elucidated in future research.

In conclusion, the ATF4/SLC1A5 axis affects glutamine metabolism and aerobic glycolysis in colorectal cancer cells, which contribute to the malignant behavior of these cells. On the other hand, our study also highlights the potential of co-targeting glutamine metabolism and glycolysis in combination with an ATF4 inhibitor.

## Supporting information

24401Supplementary_Figure_S1

## Supplementary Data

Supplementary data is available at *Acta Biochimica et Biophysica Sinica* online.
