# Supplementary material for: ATF4 promotes glutaminolysis and glycolysis in colorectal cancer by transcriptionally inducing SLC1A5: ATF4-dependent SLC1A5-mediated glycolysis
Source: Acta Biochim Biophys Sin (Shanghai). 2024 Dec 17;57(7):1093–105. doi: 10.3724/abbs.2024226 (PMC12383793; doi:10.3724/abbs.2024226)
Supplement: 24401Supplementary_Figure_S1 [file 24401Supplementary_Figure_S1.docx]

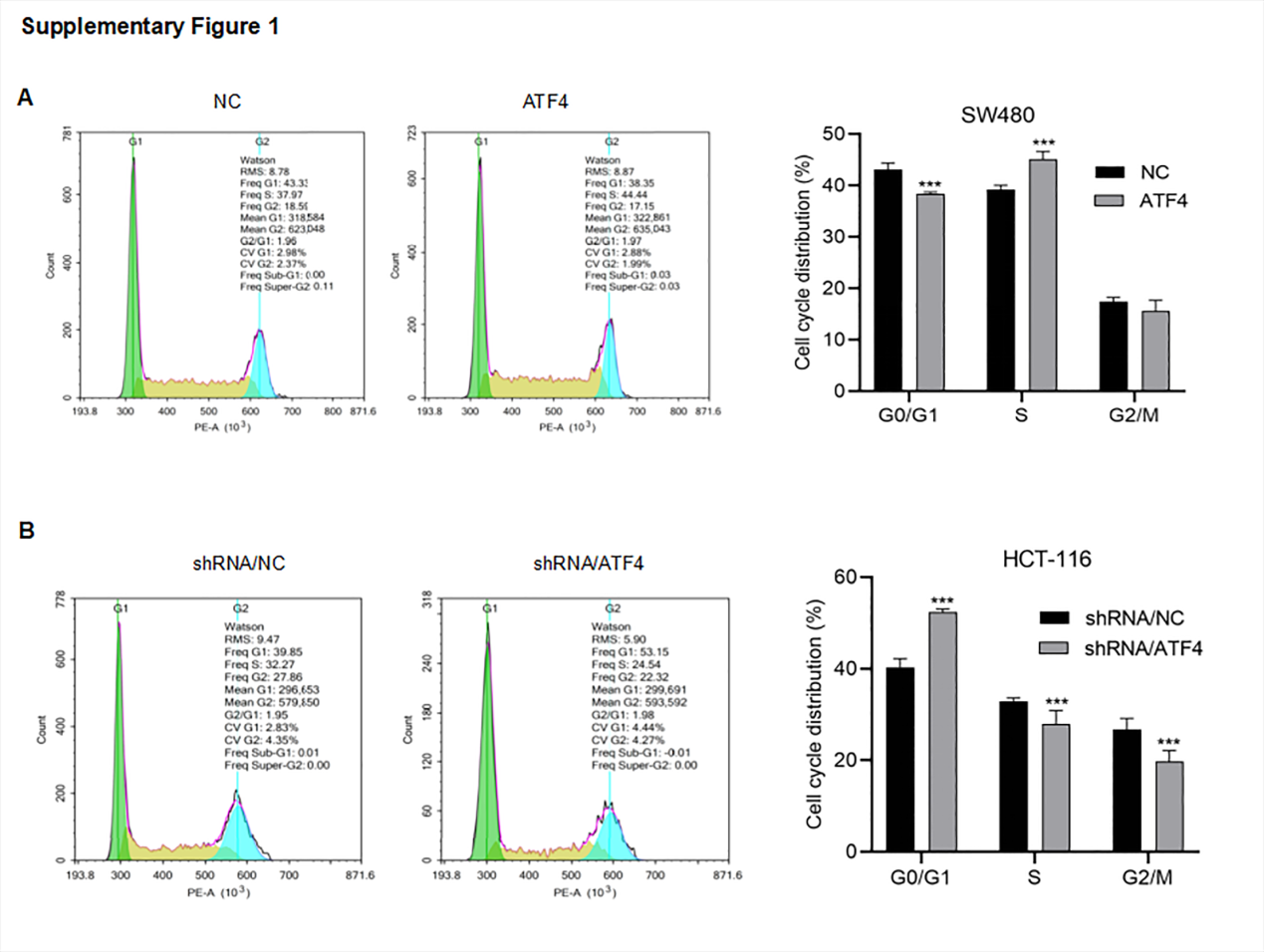


**Supplementary Figure S1. Cell cycle shift in ATF4-overexpressing SW480 cells and the inhibition of ATF4 in HCT116 cells**  (A,B) ATF4-overexpressing SW480 cells (A) and ATF4-inhibited HCT116 cells (B) were subjected to cell cycle analysis via flow cytometry after propidium iodide (PI) staining. A representative histogram of the gated cells in the G0/G1, S, and G2/M phases is presented. Quantitative analysis of the distribution of cells in each phase was performed for at least 10,000 cells per sample. Data are presented as the mean ± SEM from three independent experiments. **P*<0.05 vs control. NC, normal control.
